# Mineral Oils in Olive Oils: Background, Analytical Determination, Sources of Contamination, and Possible Mitigation Strategies

**DOI:** 10.3390/foods15081281

**Published:** 2026-04-08

**Authors:** Sabrina Moret, Seyedeh Farnaz Sadeghian, Luca Menegoz Ursol, Laura Barp

**Affiliations:** Department of Agri-Food, Environmental and Animal Science, University of Udine, via Sondrio 2/a, 33100 Udine, Italy

**Keywords:** mineral oil hydrocarbons (MOH), MOSH, MOAH, olive oil, EN ISO 20122:2024, olive pomace oil, mitigation strategies

## Abstract

Mineral oil hydrocarbons (MOH), comprising saturated (MOSH) and aromatic (MOAH) compounds, are ubiquitous lipophilic contaminants. This review critically examines their occurrence, toxicology, analysis, contamination sources, and mitigation strategies in the olive oil sector. Emphasis is placed on analytical evolution, highlighting online LC-GC-FID and the EN ISO 20122:2024 standard, including advances in saponification and epoxidation to minimize biogenic interferences. Monitoring data reveal that virgin olive oils from the market can sometimes exceed the 2.0 mg/kg limit for the MOAH. Ten times higher levels are usually found in olive pomace oils (OPOs). In OPO, solvent extraction causes a significant reconcentration of hydrocarbons remaining on the solid matter after physical extraction and accumulating during the open-air storage of pomace. Conversely, for virgin oils, contamination can occur at multiple points along the supply chain, but harvesting emerged as the most important critical step, often due to accidental contact with lubricants, greases, or hydraulic fluids. Post-milling operations may also contribute to contamination. Mitigation strategies rely on Good Agricultural and Manufacturing Practices, focusing on the systematic replacement of technical-grade lubricants with food-grade alternatives. Additionally, olive washing can reduce initial MOSH content, while refining further lowers levels, particularly in lighter fractions.

## 1. Introduction

Mineral oil hydrocarbons (MOH) are a class of ubiquitous environmental and processing contaminants, commonly present in different food matrices at mg/kg levels. Their main origin is petrogenic, and therefore attributable to crude oil and products obtained from the distillation and subsequent refining/purification of petroleum. The preponderant fraction is the saturated hydrocarbon fraction called MOSH (mineral oil saturated hydrocarbons), including linear, branched, and cyclic compounds. This is accompanied by a variable percentage (usually 0–30%) of the aromatic hydrocarbon fraction, called MOAH (mineral oil aromatic hydrocarbons), composed of 1–7 aromatic rings with different degrees of alkylation [1,2].

Analytically, MOSH and MOAH are typically quantified as unresolved complex mixtures [3], as further detailed in Section 2.1.

The extent of contamination depends on both the source and the composition of the food. Indeed, food matrices such as edible oils and fats are more susceptible to MOH contamination due to their high chemical affinity for non-polar contaminants. Consequently, vegetable oils have proven to be among the most contaminated foodstuffs, representing the primary source of dietary exposure to MOH according to the European Food Safety Authority (EFSA) [1,2,4,5,6]. This is particularly relevant for the Mediterranean diet, where the high and frequent consumption of olive oil significantly contributes to increasing human exposure to this class of contaminants. Although this review focuses on the olive oil sector, many of the analytical challenges and mitigation strategies discussed are applicable to other vegetable oils and fats.

**Figure 1:**
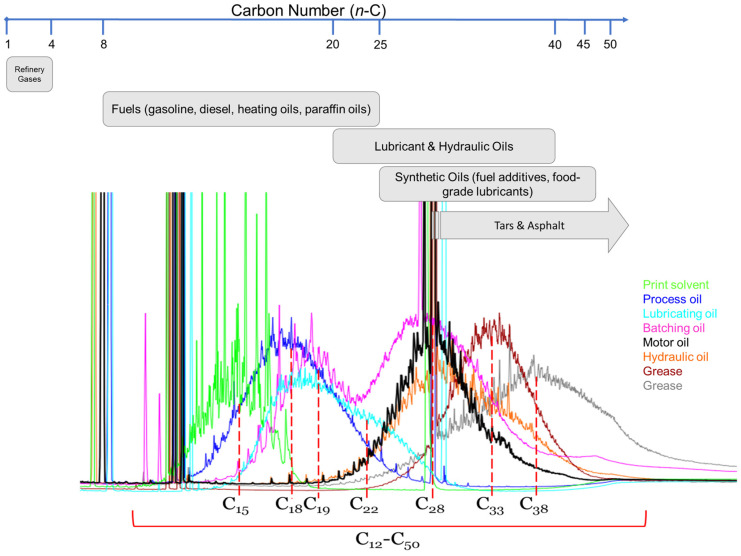
Carbon number (*n*-C) distribution of the main classes of MOH, including their typical contamination sources and representative LC-GC-FID chromatograms. The range from *n*-C_10_ to *n*-C_50_ highlights the current analytical window for monitoring and data reporting in edible oils according to the latest Joint Research Centre (JRC) and European Food Safety Authority (EFSA) guidelines [2,11].

The ubiquitous presence of these contaminants stems from the widespread use of petroleum-derived products across multiple sectors. Lighter fractions include refinery gases (*n*-C_1_-C_4_), followed by fuels like gasoline, diesel, and heating oils, which generally contain hydrocarbons from *n*-C_8_ to *n*-C_25_ and a high proportion of *n*-alkanes. Within this range also fall paraffin oils, often used for plant protection purposes. Intermediate fractions include lubricating (e.g., motor oils) and hydraulic oils, typically centered around *n*-C_23_-C_30_ and distributed in the range *n*-C_20_-C_40_. These, as well as heavier products, are generally deparaffinated over *n*-C_22_ to avoid wax crystallization [7,8]. Heavier fractions include greases, centered around *n*-C_30_-C_35_, and synthetic oils (e.g., fuel additives or food-grade lubricants) ranging from *n*-C_25_ to beyond *n*-C_45_. Finally, residues reaching over *n*-C_50_ are found in tars used for asphalt production [9,10] (Figure 1).

It is therefore evident that the sources of contamination can be manifold, either voluntary or accidental, and can occur at almost any stage of vegetable oil production. Oils can be contaminated with unrefined or partially refined MOH (containing both MOSH and MOAH) or food-grade MOH, from which MOAH have been largely removed [3,4,6].

In recent years, MOH contamination in olive oil has emerged as a significant concern for food safety and quality assurance. Beyond compromising purity and sensory qualities, these contaminants pose potential health risks due to their bioaccumulative nature and potential carcinogenicity. As olive oil is globally valued for its health benefits, understanding the sources, detection methods, and mitigation strategies is crucial to ensure product integrity and public health. Addressing this issue requires a multidisciplinary approach involving analytical chemistry, food regulation, and industry best practices.

### 1.1. MOH Toxicity and Public Health Concerns

The toxicological relevance of MOSH is strictly dependent on their chemical structure and molecular weight. MOSH are known to accumulate in human tissues [12], including the liver, spleen, and lymph nodes, as well as in human milk [13]. Evidence suggests that humans accumulate MOSH to higher levels than rats, showing a marked preference for heavier hydrocarbons [14]. According to the updated EFSA Opinion [2], current dietary exposure to MOSH does not pose short- or medium-term health risks for any consumer group. Nevertheless, long-term effects remain a subject of investigation, particularly regarding the observed increases in liver weight in animal models. Special attention is directed toward alkylated cycloalkanes, which are poorly metabolized and thus exhibit a higher potential for bioaccumulation [2,14,15].

The MOAH fraction presents a more complex toxicological profile. MOAH, consisting of 1–2 rings, are not classified as genotoxic carcinogens but may act as co-carcinogens; consequently, EFSA has recommended the collection of more robust data regarding their oral toxicity. In contrast, compounds containing three or more benzene rings with minimal or no alkylation are generally considered genotoxic carcinogens. EFSA has emphasized the urgent need for data on how ring alkylation influences the toxic potential of this fraction, alongside the development of advanced analytical methods to isolate and quantify these specific compounds [2,16].

As vegetable oils often contain the highest concentrations of MOH among food categories, they represent a primary contributor to dietary exposure. However, thanks to the mitigation efforts implemented in recent years, the 2023 EFSA Opinion reported a general decrease in exposure levels across all population groups, reflecting improvements in industrial practices and monitoring [2].

### 1.2. MOH in Olive Oils: Critical Monitoring and Occurrence Data

As reviewed by Brühl [17], MOH levels in edible oils vary significantly depending on exposure to contamination sources and the extraction technologies employed. While saturated hydrocarbon analysis has been established for decades, standardized methods for the routine determination of MOAH only became available after 2009 [18].

A major challenge in assessing MOH occurrence in olive oils, particularly virgin ones, is the presence of endogenous compounds. The co-elution of natural olefins (e.g., squalene and its isomers) can lead to significant overestimation of the MOAH fraction if not properly removed during sample cleanup. Similarly, endogenous *n*-alkanes can interfere with the MOSH humps. Therefore, the evolution of data reflects not only changes in contamination but also the increasing efficiency of purification techniques, culminating in the recent ISO 20122:2024 standard [19].

#### 1.2.1. Evolution of Data Reporting and Monitoring

Historically, results were expressed as the sum of fractions up to *n*-C_35_, following early toxicological models [1]. However, evidence of human bioaccumulation up to *n*-C_50_ [12] shifted the analytical window. Following the first Joint Research Center (JRC) Technical Report, the analytical consensus evolved toward reporting the sum of specific fractions up to *n*-C_50_ [11]. The updated 2023 JRC guidance now mandates reporting total hydrocarbon content (MOSH or MOAH) over the range *n*-C_10_–C_50_ [20].

According to EFSA monitoring (2011–2021), the average MOSH levels were 10 mg/kg for olive oils (including virgin, extra virgin, and refined categories, *n* = 299) and 108 mg/kg for olive pomace oil (OPO, *n* = 51). MOAH data remained “left-censored” (92% below the limit of quantification, LOQ), with mean concentrations between 0.3 mg/kg (lower bound) and 1.2 mg/kg (upper bound) for olive oils, and 14 mg/kg for OPO [2].

#### 1.2.2. Occurrence

Table 1 reports the total MOSH and MOAH (*n*-C_10_-C_35_) obtained by Luisi [21] from a wide range of extra virgin olive oils (EVOOs) of different origins, analyzed in 2018.

Non-EU EVOOs exhibited the lowest average MOSH *n*-C_10_-C_35_ concentrations at 4.4 mg/kg, whereas EVOOs originating from Greece showed the highest levels, reaching 18 mg/kg. Concerning MOAH, the highest average values were observed in samples from Spain (4.9 mg/kg), with Greek EVOOs following closely at 3.9 mg/kg. As discussed in detail in Section 3.3 and Section 3.4, these regional differences are probably due to variations in pruning, harvesting, and/or processing practices. More recent literature data (2019–2025) for total MOH (*n*-C_10_-C_50_) are summarized in Table 2. This table also includes information regarding sample origin (when known), the analytical method used, the declared LOQ, and the number of samples analyzed.

When comparing data from different sources, it is important to emphasize that discrepancies may occur due to the different LOQ values applied, especially when calculating total concentrations as the sum of individual C-fractions. Indeed, before the standardized approach consolidated around 2023, the LOQ often referred to individual C-fractions rather than the total sum, leading to inconsistencies, especially in reporting low-level contaminations.

MOSH (*n*-C_10_-C_50_) levels reported for 177 EVOOs sampled in 2019 ranged from the LOQ (1.0 mg/kg) up to 194 mg/kg (average 13 mg/kg), whereas MOAH exceeded LOQ (1.0 mg/kg) in 56% of the samples (average 2.2 mg/kg) [21].

When considering historical data (dating back to 2003 and reported in [1]), the concentrations of MOSH in EVOO samples show a certain consistency in terms of occurrence, with values typically ranging from <1 to 11 mg/kg (*n* = 12). A similar trend is observed for crude OPO (COPO), which consistently exhibits the highest contamination levels, with average MOSH values reaching 230 mg/kg (*n* = 3).

These findings are further supported by the average total MOSH and MOAH (*n*-C_10_-C_50_) levels recently reported by Menegoz Ursol et al. [22]. In their study, 22 EVOO samples from the market showed average concentrations of 19.4 and 3.4 mg/kg, respectively. Interestingly, within that specific sample set, conventional EVOOs showed higher average MOSH levels (23.0 mg/kg) compared to organic ones (15.0 mg/kg), while MOAH levels remained comparable (3.3–3.5 mg/kg) [22]. To the best of our knowledge, no other studies currently differentiate between these two farming systems in terms of MOH contamination. Therefore, these findings should be interpreted as a preliminary trend based on a limited market survey, rather than a definitive statistical characterization.

Overall, EVOO samples from the market showed higher contamination than those collected directly from the centrifuge exit at the mill (*n* = 25), which averaged 8.6 mg/kg for MOSH and 1.7 mg/kg for MOAH [22]. This discrepancy (19.4 mg/kg in market samples versus 8.6 mg/kg at the mill) suggests that post-milling operations (e.g., storage, filtration, and bottling) represent further critical contamination steps [22].

When comparing different categories, olive oils (OOs, a blend of refined *lampante* and virgin olive oils) from the market exhibited higher contamination levels than refined olive oils (ROOs) or the original *lampante* olive oils (LOOs) [24,26]. The deodorization phase during refining typically reduces contamination, especially for the fraction below *n*-C_25_. However, the elevated levels found in commercial blends suggest that additional post-refining sources, such as storage and bulk transport, may also contribute to the final MOH contamination.

As consistently shown in Table 1, OPO is the most critical matrix within the olive sector. Its extreme contamination, frequently exceeding 100 mg/kg, is not merely accidental but systemic, arising from the unique nature of its production cycle (as discussed in Section 4). OPOs from Spain showed lower contamination levels, with an average of 106 mg/kg of MOSH and 13 mg/kg of MOAH. In contrast, OPOs from Italy exhibited higher averages of 170 mg/kg and 31 mg/kg, respectively. This difference may arise because the Italian COPOs were all solvent-extracted, whereas the Spanish samples included products obtained through physical extraction [25].

Regarding the temporal evolution of MOH contamination in olive oils, the literature currently lacks systematic longitudinal studies. Future research should focus on consistent monitoring programs using standardized protocols to objectively evaluate the long-term effectiveness of the recently implemented mitigation practices along the entire production chain.

### 1.3. Regulatory Limits

To date, no legally binding maximum levels (MLs) for MOSH and MOAH have been fully implemented in the European Union for vegetable oils. However, the regulatory landscape has undergone significant evolution over the last two decades.

The first major “alarm” occurred in 2008, following the high-level contamination of crude sunflower oil originating from Ukraine [27,28]. At that time, a temporary limit of 50 mg/kg for paraffinic hydrocarbons was established to manage the crisis. However, it was later withdrawn as the focus shifted toward a more comprehensive toxicological evaluation by EFSA [1].

A pivotal turning point occurred in April 2022, when the European Standing Committee on Plants, Animals, Food and Feed (SCoPAFF) issued a joint statement. This document recommended the immediate withdrawal of food products from the market when the sum of MOAH concentrations exceeded specific action levels, based on the LOQs established by the JRC. For vegetable oils (fat content > 50%), this threshold was set at 2.0 mg/kg. Although not a formal regulation, this statement has de facto acted as a limit for many national control authorities and retail chains.

By the end of 2023, the European Commission initiated the process to include formal maximum levels for MOAH within Regulation (EU) 2023/915 [29] concerning contaminants in food. According to the latest draft revisions (expected to be finalized at the beginning of 2027), a maximum limit of 4.0 mg/kg is proposed for virgin and EVOOs, with a further reduction to 2.0 mg/kg scheduled for January 2028. Recognizing the distinct technological challenges of the OPO sector, the draft proposes a more gradual roadmap, starting with a limit of 10.0 mg/kg and scheduled to reduce to 2.0 mg/kg by 2030.

Regarding MOSH, despite their high bioaccumulation potential, no specific limits are currently included in the upcoming EU draft regulation. While official harmonized limits are still pending, the European market is heavily influenced by voluntary industry standards. A prime example is provided by the German Food Association (Lebensmittelverband Deutschland), which recently updated its orientation values (August 2024), setting a benchmark for MOSH in vegetable oils at 13.0 mg/kg [30].

## 2. Analytical Determination

Vegetable oils are particularly challenging matrices for MOH analysis due to the typically low concentration of contaminants relative to the dominant triglyceride matrix. Additionally, numerous endogenous compounds co-extracted during sample preparation can co-elute with the target analytes, significantly complicating accurate quantification. Consequently, precise determination requires a sophisticated balance between high sensitivity and effective removal of these interferences, making sample preparation the decisive stage for reliable quantification [31,32].

### 2.1. The Reference Method: Online LC-GC-FID and the ISO 20122:2024

The determination of MOH is currently based on online high-performance liquid chromatography coupled to gas chromatography with flame ionization detection (HPLC-GC-FID). This technique was originally introduced by Biedermann and Grob [18] and subsequently improved through the addition of an enrichment step based on saponification. This refined approach is now the officially recognized method for MOH analysis [2].

In this system, the HPLC unit, typically equipped with a silica column, fractionates the sample extract into MOSH and MOAH. The two fractions are transferred online to the GC via a dedicated interface and quantified by FID as distinct groups. In the chromatograms, they appear as broad humps corresponding to unresolved complex mixtures. Accurate quantification and control of potential analyte losses are ensured through the use of a specific internal standard (IS) mixture. For the MOSH fraction, standards typically include *n*-C_11_, *n*-C_13_, cyclohexyl cyclohexane (CyCy), and cholestane; for the MOAH fraction, pentylbenzene (PB), tri-tert-butylbenzene (TBB), 1- and 2-methylnaphthalene (1-MN and 2-MN), and perylene (Per) are commonly used. These compounds enable the correction of final results based on recovery and define the retention time windows [3,18]. To minimize analytical uncertainty and ensure data comparability, these protocols are typically integrated with rigorous quality control practices, including regular blank monitoring.

A major advancement in method performance was achieved with the transition from EN 16955:2017 [33] to the more sensitive EN ISO 20122:2024 [19]. The former relied on fat retention onto the LC silica column (physical adsorption) for the removal of the triglyceride matrix, which limited the maximum amount of sample that could be processed to 20 mg. This resulted in a relatively high LOQ (10 mg/kg) when calculated based on interlaboratory standard deviation. The revised ISO standard formally introduced saponification as an enrichment step, allowing the treatment of larger sample amounts and significantly lowering the LOQ.

The harmonized ISO 20122:2024 [19] applies to hydrocarbons in the *n*-C_10_–C_50_ range and has been validated for MOSH concentrations above 3 mg/kg. Although FID remains the detector of choice for quantifying unresolved complex mixtures due to its nearly universal and mass-proportional response, it lacks structural selectivity [34]. Therefore, comprehensive two-dimensional gas chromatography (GC×GC) is increasingly used as a confirmatory technique to complement LC-GC-FID. According to the latest EFSA Opinion [2], GC × GC is essential for verifying the MOH origin of the detected hydrocarbons. This technique allows for their distinction from biogenic interferences, such as squalene or plant waxes. Furthermore, it enables a more detailed toxicological characterization of the MOAH fraction by separating compounds based on the number of aromatic rings.

Despite the existing guidelines and initial efforts to harmonize matrix-specific protocols [11], some significant structural gaps still hinder the path toward full reproducibility of results. One of the most pressing issues is the current absence of certified reference materials, which lack the necessary tools to calibrate systems or verify method performance in real-world samples accurately [31].

Furthermore, while the analytical process has historically been hampered by a reliance on manual data processing, significant strides have been made toward full automation. The integration of online LC-GC-FID systems with advanced robotic platforms for sample preparation has drastically reduced human intervention and improved reproducibility. Recent developments have also addressed the challenge of data interpretation. Manual integration was previously seen as a primary obstacle to reducing measurement uncertainty below the 20% threshold [31,35]. However, the implementation of sophisticated software for automated integration and data handling has significantly mitigated this subjectivity [36].

### 2.2. Saponification: Essential Steps and Sources of Variability

**Figure 2:**
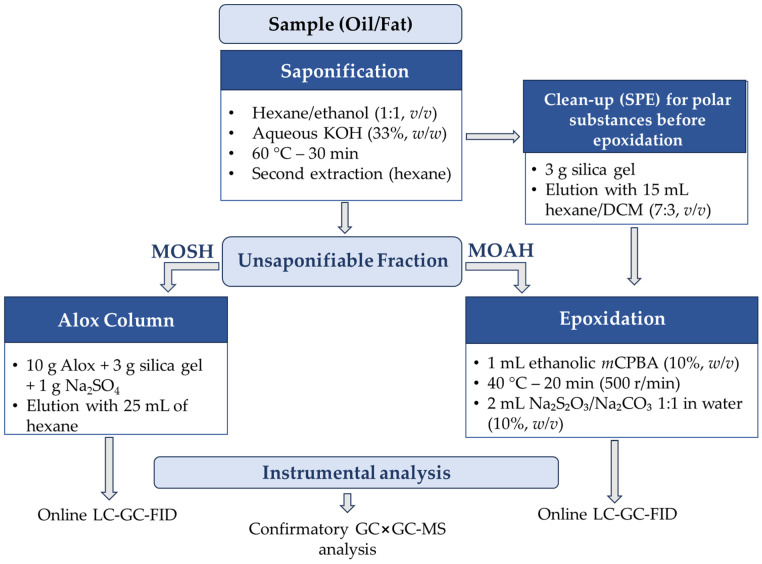
Schematic representation of the analytical workflow for MOH determination according to the ISO 20122:2024 standard [19]. DCM: dichloromethane; Alox: aluminium oxide; *m*CPBA: *m*-chloroperbenzoic acid.

In vegetable oils, the amount of sample that can be injected into a 2 mm × 250 mm silica gel HPLC column is limited to approximately 20 mg by the column’s capacity toward triglycerides [3]. Saponification is therefore an essential first step in the ISO 20122:2024 protocol (Figure 2) to reduce the fat matrix and make the unsaponifiable fraction accessible [19].

The ISO 20122:2024 standard [19] prescribes a conventional saponification in a water bath at 60 °C for 30 min using aqueous KOH, followed by manual liquid–liquid extraction. While this provides a harmonized reference, it is a significant source of variability (15–25% for MOAH), partly due to the complex handling steps and the unequal partitioning of ISs between the hydroalcoholic and organic phases [37]. Notably, while the ISO method established the benchmark, it does not explicitly include microwave-assisted protocols, which are currently considered optimized internal alternatives for high-throughput laboratories.

In fact, to improve reproducibility and efficiency, microwave-assisted saponification (MAS) has been extensively explored. In this approach, the oil sample, the alkaline solution (KOH), and the organic solvent (*n*-hexane) are introduced into a sealed vessel and heated (typically at 120 °C for 20 min). This configuration allows for simultaneous saponification and extraction: as the triglycerides are hydrolyzed, the MOH are immediately partitioned into the organic phase, minimizing handling and contamination risks.

Over the years, protocols have been progressively refined to address matrix-specific challenges. Early MAS protocols used methanolic KOH [38], but recent advancements have replaced methanol with a 2 M KOH solution in ethanol/water (1:1, *v*/*v*) [37,39]. Combined with a second hexane extraction and optimized washing strategies, this modification has stabilized the TBB/2MN ratio to 1.05 ± 0.01 across diverse fats and oils, ensuring that the simultaneous extraction is robust even for challenging matrices like fully hydrogenated fats.

### 2.3. Strategies to Remove Biogenic Interferences

Accurate quantification is often compromised by the presence of co-extracted biogenic compounds, particularly olefins in the MOAH fraction and long-chain *n*-alkanes in the MOSH fraction.

#### 2.3.1. Olefin Removal Before MOAH Determination

The presence of biogenic olefins, such as squalene (with average concentrations of 5 g/kg in olive oils [40]) and sterenes, represents the most significant analytical interference in MOAH quantification. These compounds, if not removed, elute in the MOAH window, creating “false humps” or overloading the GC column.

For this reason, dedicated epoxidation protocols should be applied before LC-GC-FID analysis to selectively oxidize olefinic compounds. The resulting epoxides exhibit a significantly higher affinity for the silica gel stationary phase during the LC fractionation step; consequently, they are retained on the column (or removed by backflush), while the non-polar MOAH fraction elutes without interferents [18].

The first epoxidation protocol involved the treatment of approximately 300 mg of oil with *m*-chloroperbenzoic acid (*m*CPBA) dissolved in dichloromethane under cooling, followed by neutralization and washing steps [18]. Although effective, this approach requires careful control to ensure complete olefin removal while preserving oxidation-sensitive aromatic compounds within the MOAH fraction [32,41,42].

**Figure 3:**
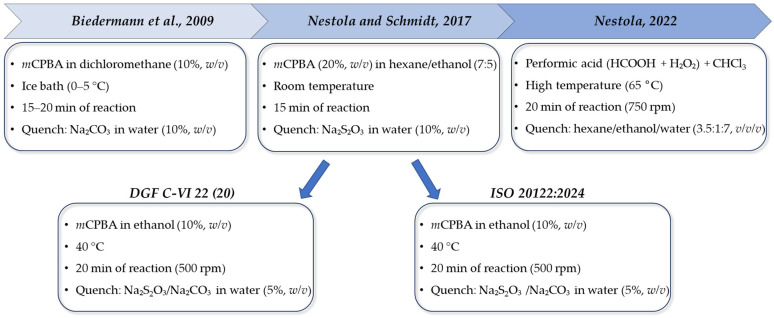
Schematic representation of epoxidation protocols according to Biedermann at al., 2009 [18], Nestola and Schmidt, 2017 [43], and Nestola, 2022 [36]. The second protocol was the base, after minor modifications, for the method proposed by the German Society of Fat Sciences (DGF) [44] and the ISO 20122:2024 standard [19].

Over time, the procedure has been refined to address practical and technical limitations of the original method (Figure 3). Nestola and Schmidt [43] proposed the use of *m*CPBA dissolved in a *n*-hexane/ethanol mixture (7:5, *v/v*). In this system, ethanol acts as a radical scavenger, moderating the reaction rate and allowing the process to be conducted at room temperature rather than under ice cooling. This modification improved analytical selectivity and aligned with Green Analytical Chemistry principles by replacing chlorinated solvents with less hazardous alternatives, while maintaining efficient olefin removal. In addition, the carbonate washing step was replaced by sodium thiosulfate quenching, which immediately stops the reaction and prevents over-oxidation during autosampler residence time [42].

Both the DGF C-VI 22 (20) [44] method proposed by the German Society of Fat Sciences and the ISO 20122:2024 [19] method are based on this protocol with minor modifications. The procedure involves epoxidation of the MOAH fraction using *m*CPBA dissolved in ethanol at 40 °C. To ensure reagent deactivation (quenching), a mixture of sodium thiosulfate and sodium carbonate (1:1) is added. Under these standardized conditions, minor peaks from peracid-related by-products may occasionally appear, but these can be minimized by appropriate purification of the reagent. Despite this harmonized cleanup, residues of highly resistant olefins may still persist, potentially contributing to background signals within the MOAH fraction [19,43].

Finally, for refined oils containing “resistant” olefins (e.g., sterenes or carotenoid degradation products), the use of performic acid generated in situ (from formic acid and hydrogen peroxide) has proven superior [36]. This reagent is more polar than *m*CPBA and operates at slightly elevated temperatures (65 °C), providing a deeper cleanup for complex matrices, such as palm oil or highly refined olive oils, while significantly reducing the “reagent blank” signals often caused by *m*CPBA impurities.

#### 2.3.2. MOSH Cleanup: Activated Aluminium Oxide

Biogenic paraffins (predominantly odd-chain *n*-alkanes in the *n*-C_23_-C_33_ range) are natural constituents of olive cuticular waxes. In GC analysis, these compounds appear as intense, sharp “riding peaks” on top of the MOSH hump. Their selective removal is essential to prevent overestimation of the petrogenic content and to ensure reliable signal integration.

The introduction of activated aluminium oxide (Alox) represented a significant improvement in cleanup strategies aimed at eliminating these biogenic interferents. The mechanism relies on the selective retention capacity of Alox activated at high temperature (400 °C), which preferentially adsorbs long-chain *n*-alkanes (>*n*-C_20_), while branched (iso-alkanes) and cyclic saturated hydrocarbons, representing the majority of petrogenic MOSH, elute with minimal retention [45].

Early approaches [33,45] involved direct passage of the solvent-diluted oil through large Alox columns or mixed Alox/silica beds. These configurations required substantial amounts of stationary phase (up to 20 g of Alox) and correspondingly high solvent volumes (up to 40 mL of *n*-hexane).

The ISO 20122:2024 method [19] proposes a mixed cleanup column containing 10 g of Alox, 3 g of silica, and 1 g of anhydrous sodium sulfate. In this case, removal of biogenic *n*-alkanes is performed after saponification as an optional step for matrices rich in biogenic paraffins, such as virgin olive and sunflower oils. This procedure still requires relatively high solvent volumes (up to 25 mL of *n*-hexane).

More recent developments have focused on reducing both the stationary phase amount and solvent consumption. Following saponification and epoxidation, effective cleanup can be achieved using only 2.5 g of activated Alox and 5 mL of *n*-hexane, significantly improving procedural efficiency while maintaining selectivity [24].

### 2.4. New Frontiers: Selective Separation and Characterization

To align with the toxicological requirements discussed in Section 1.1, recent analytical efforts have focused on differentiating MOAH by the number of aromatic rings (1-2R vs. ≥3R) [2].

#### 2.4.1. Emerging Chromatographic Strategies

Recent attention has shifted toward chromatographic approaches that structurally differentiate MOAH classes to align with these toxicological requirements.

Donor-acceptor complex chromatography (DACC) enables the automated separation of mono-/di-aromatics from tri- and polyaromatics. While effective in reducing biogenic interference, it still faces challenges with “border-crossing” compounds that may co-elute across fractions [46].

More recently, Gorska et al. (2025) [47] proposed a normal-phase HPLC fractionation on silica that allows for the separation of 1-2R from ≥3R MOAH. This configuration is particularly attractive for routine use as it utilizes the same stationary phases already present in standard LC-GC systems, avoiding the need for dedicated DACC columns, difficult to find on the market.

#### 2.4.2. Comprehensive Two-Dimensional Gas Chromatography (GC × GC)

While LC-GC-FID remains the gold standard for quantification, it lacks the resolving power to qualitatively characterize the MOAH fraction. In this context, GC × GC, typically coupled with time-of-flight mass spectrometry (ToFMS), has emerged as the most powerful tool for “confirmatory” analysis [48,49,50,51].

The primary advantages of GC × GC in olive oil analysis include the physical separation of residual biogenic olefins from the MOAH humps, identifying “false positives” that survive epoxidation. Furthermore, GC × GC is essential for addressing the emerging concern of endogenous MOAH-like compounds. Recent studies suggest that some vegetable matrices may contain natural aromatic structures (e.g., specific terpenoids or plant-derived hydrocarbons) that are not of petrogenic origin but can be misidentified as MOAH.

By separating compounds based on two different properties (volatility and polarity), it creates a structured chromatogram where MOAH are grouped by the number of aromatic rings into distinct horizontal bands, fulfilling recent EFSA requirements

#### 2.4.3. Selective Enrichment via Liquid–Liquid Partitioning

A significant limitation of current total MOAH analysis, both in mono- and bi-dimensional GC, is the masking effect caused by highly alkylated 1-2R compounds, which are often the most abundant aromatic fraction in olive oils. Their broad chromatographic signal can obscure the presence of the more toxicologically relevant ≥3R fraction, especially when the latter is present at trace levels.

To address this, recent research [52] is focusing on a targeted sample preparation strategy based on selective liquid–liquid partitioning. By optimizing the solvent system, it is possible to enrich the ≥3R MOAH fraction, which has a higher affinity for specific polar solvents compared to the more lipophilic highly alkylated 1-2R species. In this way, it is possible to reduce the background interference of the 1-2R hump, thereby lowering the LOQ for the potentially genotoxic aromatics.

This partitioning approach represents a strategic evolution in MOH analysis. It aligns with the EFSA request for more robust data on the oral toxicity of MOAH by providing a purified extract that is not only easier to quantify but also more representative of the actual toxicological risk associated with the oil sample.

## 3. MOH Sources Along the Virgin Olive Oil Supply Chain

The presence of MOH in olive oil is the result of a complex interplay between environmental deposition and operational factors throughout the production chain. In addition to primary sources, specific variables such as equipment maintenance, processing conditions, and raw material quality significantly influence the final MOH level.

Equipment maintenance is a critical variable; for example, worn seals in mechanical harvesters or centrifugal decanters can cause direct leakage of technical oils into the product. Furthermore, processing conditions, specifically malaxation temperature and duration, can affect the partition of hydrocarbons between the aqueous and oily phases. Finally, olive quality plays a dual role. Overripe or damaged fruits, often associated with higher soil residues and prolonged storage, are more prone to accumulating environmental MOH compared to high-quality, freshly harvested olives.

The systematic implementation of manufacturing practices is therefore essential to monitor and mitigate these sources of contamination.

### 3.1. Environmental Contamination

MOSH presence in the environment is ubiquitous and primarily linked to industrial and vehicular emissions and/or lubricant dispersion. Deposition from the air can occur directly from the gas phase for lighter compounds (up to *n*-C_25_) or via atmospheric particulate matter for the heavier ones. Air pollution-related contamination is facilitated by the presence of waxes on seed and fruit surfaces, which act as natural absorbers of environmental hydrocarbons due to their chemical affinity. While MOSH in the air are easily transferred to the crops, MOAH probably undergo oxidation, reducing their persistence [10,26].

Neukom et al. [10] detected mineral paraffins in the *n*-C_20_-C_50_ range across both urban and rural environments, linking their composition directly to soil and crops. They identified three primary sources characterized by increasing molecular weight: heating and diesel oil residues, lubricating oils, and asphalt. Lubricating oils were found to be the predominant contributor, as later confirmed by Grundböck et al. [53], who detected 2.4 mg/kg of MOSH in Swiss sunflower oil from seeds hand-harvested near urban areas, levels four times higher than those from rural regions.

Moret et al. [26] studied hydrocarbons collected from vehicle exhaust, finding that farm tractor emissions contained high-boiling diesel residues indicative of incomplete combustion. The study also showed that older petrol engines without catalytic converters and petrol-powered motorcycles emitted hydrocarbons directly derived from motor oil. Brandenberger et al. [54] further examined how engine conditions affect particle-bound emissions, noting that cold starts and lower temperatures significantly increased paraffin output due to inefficient combustion. Conversely, higher engine loads reduced light paraffin emissions but increased those derived from lubricating oil, shifting the profile toward higher molecular weights.

Despite this evidence, the impact of environmental contamination on total MOSH levels in olive oils appears relatively low. Gharbi et al. [55] analyzed a set of Tunisian olives hand-picked from trees (*n* = 11), reporting an average MOSH concentration of 2.6 mg/kg in the extracted oil. In all the samples, MOAH levels remained consistently below the LOQ. No correlation was found between the groves’ proximity to contamination sources (such as roads or industrial areas) and the detected MOSH levels.

Further research on oil extracted from hand-picked olives in Italy, Croatia, and Greece confirmed that, with few exceptions, MOSH levels remained below 2.0 mg/kg and MOAH below 0.5–2.0 mg/kg (detection limit), regardless of nearby contamination sources [24,56]. For example, in northeastern Italy’s Friuli region, olives grown near industrial steelworks showed only slightly higher contamination than those from rural areas. Interestingly, in rural Istria, the olive variety itself influenced contamination levels: the Pendolino variety exhibited slightly higher concentrations due to its larger surface-to-weight ratio and higher wax content [57].

### 3.2. Phytosanitary Treatments

According to Regulation (EC) 1107/2009 [58], paraffin oils, particularly those with carbon chain distributions ranging from *n*-C_11_ to *n*-C_30_ (notably CAS numbers 64742-46-7, 72623-86-0, and 97862-82-3), are authorized as active substances in plant protection products within the European Union. These oils are employed as insecticides, acaricides, and fungicides across various crops, including olives, due to their ability to form a thin, gas-impermeable film on plant surfaces that facilitates pest suffocation. Furthermore, Regulation (EU) 1165/2021 [59] permits the use of paraffin oils in pesticides authorized for organic cultivation.

Beyond their role as active ingredients, MOH are also utilized as formulation adjuvants in phytosanitary products. They are often in combination with other active substances to enhance delivery, adhesion, and persistence on crop surfaces. Although comprehensive investigations remain limited, existing studies have identified phytosanitary treatments as potential sources of MOH contamination in edible oils, such as grape seed oil [60] and olive oil [56].

According to a monitoring conducted in Italy on samples from 15 olive groves [22], the impact of phytosanitary treatments not involving paraffin oils on MOSH levels was generally negligible. However, one sample treated with zeolite exhibited a MOSH contamination level of approximately 15 mg/kg. This was likely due to lubricant leakage from the atomizer pump or the intentional addition of MOH to improve product dispersion and adhesion. Interestingly, two producers reported using “Rogor,” a product containing 8% C_9_ aromatics, including benzene isomers, which did not leave detectable residues in the olives, most likely due to the high volatility of these specific aromatic compounds.

### 3.3. Harvesting Operations

The presence of MOH in raw materials is frequently associated with harvesting operations. To increase production volumes and contain costs, mechanized systems have been widely adopted. These include tractors, trunk shakers, and harvesters, alongside manual tools powered by pneumatic, electric, or internal combustion engines. Contamination often arises from exhaust gases and lubricant leaks from mechanical parts, engines, and hydraulic circuits. For instance, sunflower seeds collected at the outlet of a combine harvester showed MOSH contamination centered on *n*-C_17_ and *n*-C_27_. These profiles are typical of diesel and lubricating oils. In such cases, the extracted oil showed an average MOSH level of 5.6 mg/kg, reaching levels of 13.7 mg/kg [53].

Based on the study by Menegoz Ursol et al. [22], harvesting operations exert a substantial impact on the final contamination of virgin olive oils. This was confirmed by the analysis of various lubricants and greases used in harvesting machinery, which revealed that many contained significant and variable amounts of MOAH.

**Figure 4:**
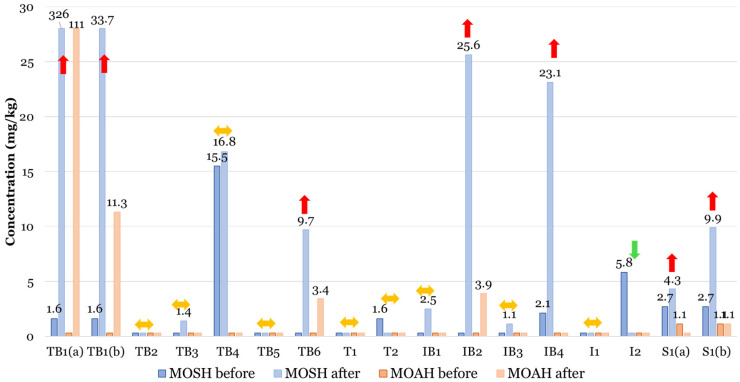
MOSH and MOAH concentrations of EVOOs obtained from olives sampled before (hand-picked directly from the trees) and after mechanized harvesting operations. Letters “T, I, S” and “B” within the sample code mean traditional, intensive, super-intensive, and biological cultivation, respectively. Absence of data labels indicates levels below the LOQ (1.0 mg/kg for MOSH and 0.5 mg/kg for MOAH). The colored arrows provide a visual summary of the concentration trends between pre- and post-harvesting: red upward arrows indicate a significant increase in concentration; yellow double-headed arrows represent negligible variations (stable levels); the green downward arrow indicates a decrease in the detected concentration. Reprinted with permission from [22]. Copyright 2022 Elsevier Ltd.

Figure 4 illustrates the MOSH and MOAH concentrations in oils extracted in the laboratory using an Abencor system. These olives were sampled across 15 Italian groves both before and after harvesting. Following harvest, a significant increase in contamination was observed in approximately 40% of the samples. Notably, 24% of these samples exceeded the proposed limit of 2.0 mg/kg for MOAH.

Of the 13 lubricants and greases provided by farmers for this study, only two were found to be free of MOH; both were used in pneumatic hand-held combs. All other products showed MOAH levels ranging from 10% to 32% (average 19%). An exception was one lubricant grease used in a straddle harvester (New Holland 2001228A, New Holland Agriculture, Turin, Italy), which contained less than 2.0% MOAH.

While the use of MOH-based lubricants and greases in harvesting machinery does not necessarily lead to olive oil contamination, it substantially increases the risk. Contamination typically occurs through accidental leaks of lubricants, hydraulic oil from pneumatic circuits, and/or direct contact with lubricated moving parts during the collection process.

**Figure 5:**
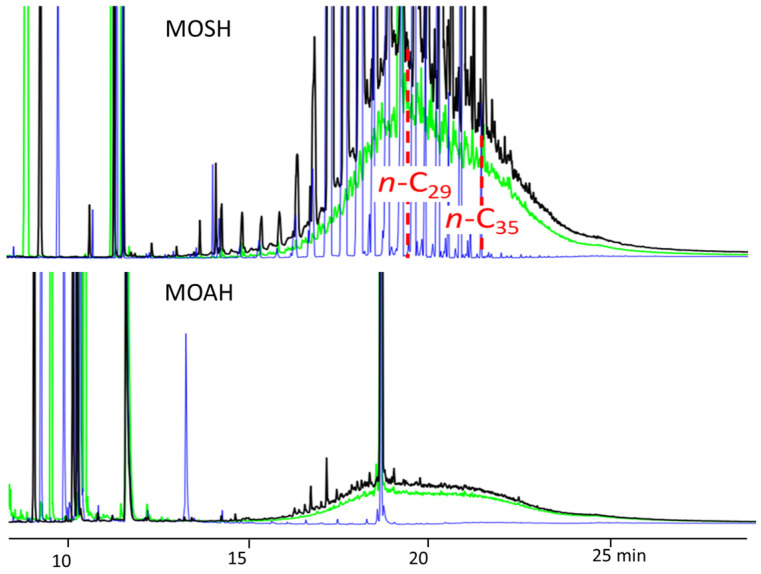
Overlay of MOSH and MOAH HPLC-GC-FID chromatograms of samples EVOO from olives sampled before (blue line) and after harvesting (black line), hydraulic oil collected from the vibrating comb (green line). Adapted with permission from [22]. Copyright 2022 Elsevier Ltd.

Figure 5 provides a representative example of this risk, displaying the LC-GC-FID traces of sample TB1(a) from an organic olive grove. While the hand-picked olives showed negligible contamination, the profile changed dramatically after harvesting with a mechanized vibrating comb. Following an accidental leak from the hydraulic circuit, the contamination levels in the extracted olive oil reached 326 mg/kg of MOSH and 111 mg/kg of MOAH.

Surprisingly, oil extracted from olives collected from the same grove (sample TB1(b)), but using pneumatic combs, exhibited an identical contamination profile, though at a tenfold lower level. Both LC-GC-FID and GC×GC-FID/MS analyses confirmed that the source was hydraulic oil, likely absorbed by the collection nets during the previous harvest and subsequently transferred to the new crop [22].

Oil extracted from olives TB6, harvested using a trunk shaker equipped with a self-propelled collection umbrella, was found to be contaminated by lubricating grease. In this case, the machine’s design allowed for direct contact between the olives and the greased mechanical components. These findings highlight the urgent need to either replace technical greases with MOH-free alternatives and/or to redesign such machinery to prevent any physical contact between the fruit and lubricated parts.

**Figure 6:**
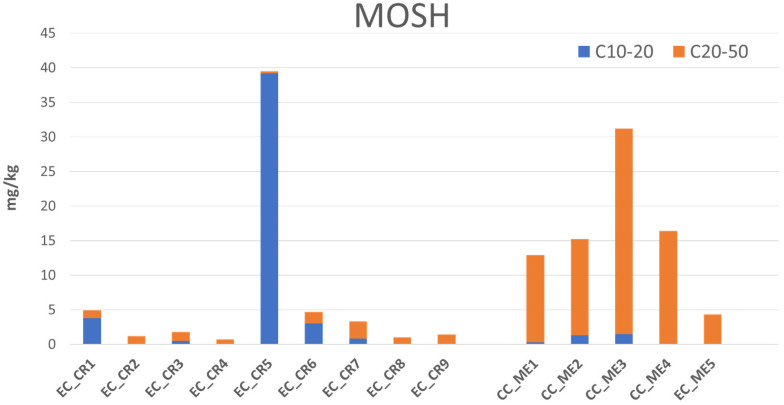
MOSH content (mg/kg) in EVOOs from two different regions in Greece: Crete (CR) and Messenia (ME), obtained from olives harvested and pruned using electric chainsaws (EC) or conventional chainsaws (CC).

In certain regions of Greece, the common practice of pruning during the olive harvest can lead to high contamination levels primarily due to the lubricants used in chainsaws, which frequently contain MOAH. Figure 6 displays MOSH levels found in olive oils from two different Greek areas with significantly different pruning practices. In Messenia, conventional chainsaws lubricated with MOH-based products are predominantly used. In contrast, electric-powered chainsaws have been more widely adopted in Crete, leading to different contamination profiles [56].

Total MOSH are visualized as the sum of two sub-fractions (*n*-C_10_-C_20_ and *n*-C_20_-C_50_) to distinguish between different contamination origins. Specifically, only the heaviest fraction can be directly related to lubricant-derived contamination. Samples from the Messenia region, which underwent pruning with a conventional chainsaw (CC), showed MOSH (*n*-C_20_-C_50_) levels between 16.1 and 29.7 mg/kg. These samples exhibited the typical chromatographic profile of chainsaw lubricants, generally centered on *n*-C_29_. In contrast, the control sample EC_ME5, pruned with an electric chainsaw (EC), showed significantly lower contamination (4.2 mg/kg). Similarly, samples from Crete, all pruned during harvest but with electric chainsaws, exhibited only background MOSH levels (<5 mg/kg), except sample EC_CR5. This particular sample showed relatively high contamination in the *n*-C_10_-C_20_ range, a profile likely attributable to a phytosanitary treatment rather than harvesting lubricants.

### 3.4. Olive Transport, Storage, and Movement Inside the Mill

In general, the transport of olives from the groves to the mill, as well as movement within the facility, does not seem to significantly affect MOH contamination. Based on monitoring results by Menegoz Ursol et al. [24], only two out of 25 cases showed a noticeable increase in contamination associated with transport or movement. Olives were primarily transported in plastic bins, typically made of high-density polyethylene (HDPE) or acrylonitrile butadiene styrene (ABS) with a capacity of approximately 500 kg, or loosely loaded into open trailers. When olives in open trailers are exposed to air and exhaust fumes, the relatively brief duration of transport generally limits the extent of contamination. Greater risks may arise if olives are moved within the mill using internal combustion forklifts instead of electric ones, as exhaust gases can accumulate in enclosed environments. However, none of the milling operators in the cited study [24] reported using combustion-powered forklifts for this purpose. Additionally, a potential risk remains from the dripping of hydraulic fluids from machinery during handling.

In some regions, such as parts of Greece, where olives are still transported in jute bags, there is a specific risk of contamination from batching oils used to treat the fibers [56]. Jute fibers are treated with these crude MOH, which typically contain both MOSH and MOAH, to enhance flexibility and performance during weaving. During storage and transport, MOH can migrate from the bags to the olives, primarily via the gas phase, followed by recondensation.

In the Greek study (data reported in Figure 6), two samples (EC_CR1 and EC_CR6) exhibited a characteristic hump in the *n*-C_16_-C_20_ range, with contamination levels of 3.3 mg/kg and 3.0 mg/kg, respectively. This contamination was likely originated from the jute bags, a hypothesis further confirmed by the presence of MOAH within the same molecular mass range (1.6 mg/kg in sample EC_CR1).

### 3.5. Processing at the Mill

Additional contamination sources can arise during the extraction process at the oil mill [61]. These include accidental contact with MOH-based products, such as lubricating and hydraulic oils, as frequently reported in the past [62]. Furthermore, the use of extraction aids, such as talc, has been investigated as a potential contamination source. Gómez-Coca et al. [63] identified paraffins in talc, which is typically employed at levels of 1–3% in olive paste to enhance extraction efficiency. Analysis of both the talc and the resulting pomace oil, derived from olive paste with (49 mg/kg) and without (27 mg/kg) talc addition, confirmed this contamination pathway. Additionally, leaks from malfunctioning press pumps have been reported to contaminate EVOOs with technical-grade oils [26].

The extraction method itself significantly influences MOH levels. Physically extracted oils tend to retain a smaller fraction of MOH. This occurs because a significant portion remains adsorbed onto the solid structures of the olive pomace, due to the limited extraction capacity of mechanical methods [26,63,64]. Conversely, solvent-assisted extraction results in substantially higher MOH concentrations, often 2 to 10 times greater, since the process achieves near-quantitative recovery of all lipid-soluble components [55,65,66].

**Figure 7:**
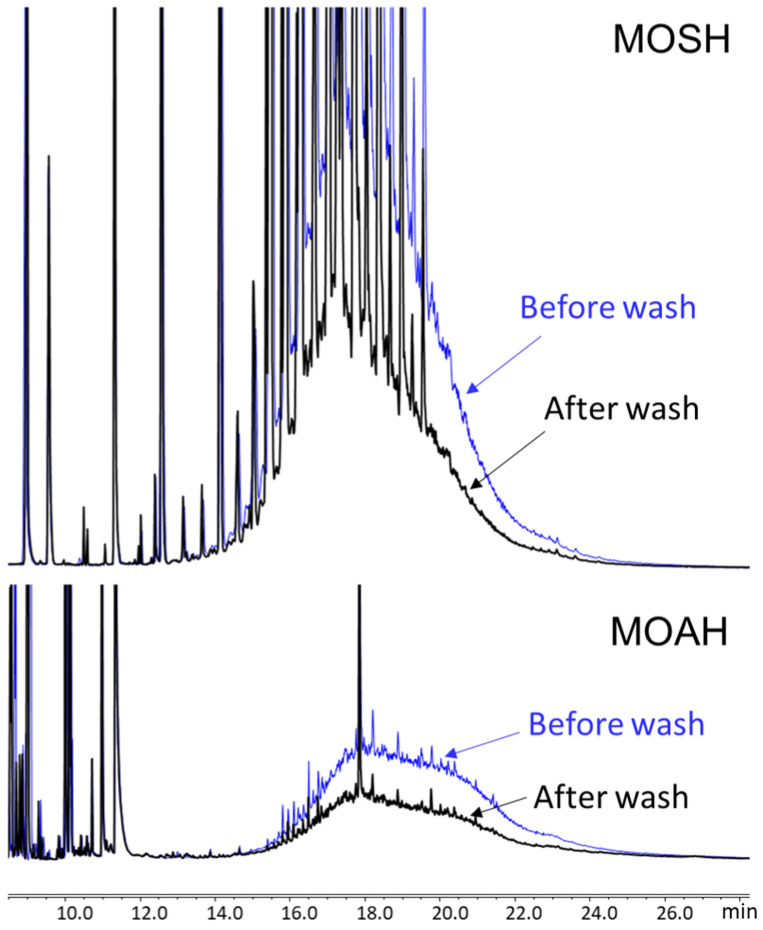
Example of MOH removal after washing (olive sample TB1).

Recently, Menegoz Ursol et al. [24] monitored 25 processing lines across five distinct stages: olives upon arrival at the mill, before and after washing, after crushing/kneading, and the final oil after vertical centrifugation. Before milling, olives are typically washed with fresh or recycled water to remove soil, dust, and residues, followed by a rinse with water jets. In this survey, washing operations showed a potential reduction in contamination in 24% of the lines investigated, particularly in samples with higher initial levels (>10 mg/kg MOSH). However, the effectiveness of this step appears highly variable, depending on the initial contamination level, its distribution, and the specific design of the washing machinery. On average, washing resulted in a mean reduction of 2.1 mg/kg in total MOSH content. Conversely, the milling process was associated with an increase in hydrocarbon levels in approximately 20% of the cases, with total MOSH and MOAH concentrations rising by an average of 2.3 mg/kg and 0.6 mg/kg, respectively [24]. An example of this effect is reported in Figure 7.

These findings, when compared with past data, show a drastic reduction in MOH contamination at the mill, indicating improved awareness among mill operators of the main sources of contamination. Indeed, most lubricants and greases used in the mills were identified as food grade (NSF NH1 certificates) or synthetic. Only two exceptions were noted: a lubricant for the oil separator containing 19% MOAH (which fortunately did not result in detectable oil contamination) and a grease not available for analysis. Both were declared as technical grade. The widespread adoption of food-grade products reflects an increasing focus on safety among mill operators. A phase-out of MOH-based lubricants in favor of MOH-free or MOAH-free alternatives represents a crucial step to further mitigate the risk of accidental contamination during the extraction process.

### 3.6. Post-Milling Operation

As reported in Section 1.2.2, the impact of post-milling operation is clearly evidenced by comparing oils sampled directly at the mill (average 8.6 mg/kg MOSH) with those collected from the market (average 19.4 mg/kg) [22]. This significant increase identifies filtration, storage, transport, bottling, and packaging materials as critical contamination points. Despite this, systematic studies on the specific impact of individual post-milling stages remain limited.

In olive oil production, filtration is a crucial final step to remove suspended solids and residual water that can cause rapid oxidation and off-flavors. While traditional systems employ filter presses with cellulose, cotton, or diatomaceous earth, newer methods utilize membrane cross-flow filtration and polypropylene filter bags. It is important to note that some of these materials, or the chemicals used for their cleaning, may themselves contain MOH residues.

Cross-contamination frequently occurs during storage and/or transportation in tanks previously used to transport contaminated product, or when oils are pumped through shared valves and pipelines. A notable case reported by Moh et al. [67] involved up to 85,000 metric tons of crude palm oil shipped to Europe from Indonesia being contaminated with diesel oil from previous cargoes. To address this risk, EFSA has evaluated the acceptability of various substances as “previous cargoes” for edible fats and oils (Commission Regulation (EU) No 579/2014 [68]).

Regarding the bottling phase, contamination can originate from contact with packaging materials or from lubricants used in the bottling machinery.

Metal containers may transfer white MOH used to protect the surface from staining or to lubricate the molds during forming [69]. Similarly, mineral waxes or oils used to treat bottle caps can contaminate the product [17,62], representing a critical point even for glass packaging. Finally, plastic materials can release polyolefin oligomeric saturated hydrocarbons (POSH), which are often indistinguishable from MOSH when analyzed via LC-GC-FID [7,55,70].

Accidental lubricant leaks from bottling equipment are a direct risk. Specifically, the air rising process, used to remove dust before filling, can be a source of contamination. Oil-lubricated compressors release vapors and aerosols that, without high-efficiency filtration (e.g., activated carbon) or proper maintenance, are inserted directly into the bottles.

Even when using oil-free compressors, ambient air that is drawn into the system can contain MOH particles. These pollutants become concentrated during compression and are subsequently transferred into the final package.

## 4. Additional MOH Sources in the OPO Supply Chain

The contamination profile of OPOs represents a unique and challenging case within the olive sector. These oils consistently exhibit MOH levels significantly higher than those found in virgin olive oils [25,26,63,71,72,73]. This disparity is not incidental but stems from a combination of mechanical, chemical, and environmental factors inherent to the OPO production cycle.

Unlike olives, which are processed shortly after harvest, olive pomace (the solid by-product of milling) is often stored in large outdoor piles for extended periods before being transported to the extraction plant. During this stage, the pomace acts as a chemical “sink” for environmental pollutants. Its high surface-to-volume ratio and porous structure facilitate the adsorption of airborne hydrocarbons and atmospheric deposition [25,40].

Several factors contribute to a steady increase in MOSH and MOAH levels during storage. These include exposure to vehicle exhaust gases from heavy machinery, oil leaks from loaders, and contact with MOH-rich dust or asphalt residues. Studies have confirmed a direct correlation between storage duration and contamination, suggesting that the porous structure of the pomace, combined with its high surface area, facilitates the absorption of airborne hydrocarbons [26,71]. Consequently, longer exposure times in storage areas significantly elevate the final MOH content.

The elevated MOH content in OPO is primarily a consequence of the solvent extraction process and the so-called reconcentration effect. During the initial physical extraction of virgin olive oil, a significant portion of lipophilic contaminants remains trapped within the solid matrix of the pomace. When this pomace is subsequently treated with organic solvents (typically *n*-hexane), these surface-bound hydrocarbons are exhaustively recovered along with the residual lipids (triglycerides). This phenomenon is driven by the preferential partitioning of MOH into the solvent phase. Since the yield of extractable oil from pomace is relatively low (typically 2.5% to 9.5%), the contaminants that remained after mechanical extraction and distributed across the large mass of the solid residue become concentrated into a much smaller lipid volume. Consequently, this dynamic leads to a dramatic enrichment: empirical data indicate that solvent-extracted oils can exhibit contamination levels on average four times higher than those found in physically extracted oils from the same batch of olives [26,71].

A comparison of MOH levels in COPO obtained through different extraction methods, namely physical extraction by centrifugation and solvent extraction, reveals a pronounced concentration effect. Experimental data indicate that MOSH levels ranged from 58 to 118 mg/kg in centrifuged samples (*n* = 7), whereas solvent-extracted samples (*n* = 10) showed concentrations between 209 and 520 mg/kg, corresponding to an approximate fourfold increase. A similar trend was observed for MOAH: concentrations ranged from 1.8 to 28 mg/kg following physical extraction and from 54 to 115 mg/kg after solvent extraction [25].

The drying phase of the pomace is a well-established critical control point for the formation of thermally neo-formed polycyclic aromatic hydrocarbons (PAHs) due to combustion gases [40,74]. However, its impact on total MOH levels appears to be less significant than the extraction process itself. This distinction is likely due to the chemical nature of the MOAH fraction typically found in OPO, which consists predominantly of highly alkylated mono- and di-aromatic compounds [25,71].

From an operational standpoint, reducing pomace storage times and using electric loaders represent feasible, low-cost interventions to limit hydrocarbon adsorption. Conversely, modifying the solvent extraction or drying units would require more significant capital investments. However, such upgrades are increasingly seen as a strategic necessity to meet the stricter safety standards and avoid product rejection in international markets.

Considering all these factors, the refining process is often insufficient to fully compensate for high initial contamination levels in OPO. This structural vulnerability makes the OPO category a primary target for urgent mitigation strategies and stricter supply chain controls.

## 5. Mitigation Strategies

It is currently not possible to eliminate MOH contamination in vegetable oils. However, the adoption of good practices throughout the entire supply chain remains essential. Combined with effective olive washing and refining (where permitted), these measures represent the most effective mitigation strategy for preventing and limiting contamination in the final product.

### 5.1. Following Good Manufacturing Practices

For virgin olive oils, the implementation of Good Agricultural and Manufacturing Practices (GAP and GMP) constitutes the only viable strategy to ensure compliance with emerging safety standards. Recognizing the complexity of the issue, several industry associations at both European and national levels have developed specific technical documents to support producers.

At the European level, FEDIOL (the EU vegetable oil and proteinmeal industry association) has established a comprehensive code of practice for managing MOH in vegetable oils and fats [75]. This document identifies “critical lubrication points” within the supply chain and mandates the use of H1-grade (food-grade) lubricants in any area where incidental contact with the product might occur. Furthermore, FEDIOL emphasizes the importance of controlling the entire logistics chain. This includes preventing leakage in harvesting machinery and strictly monitoring “previous cargoes” during bulk transport, ensuring that transportation tanks remain free from MOH residues.

Currently, FEDIOL is aligning its members with the upcoming EU Contaminants Regulation (updating EU 2023/915 [29]), which is expected to officially codify maximum levels for MOAH in vegetable oils by 2027.

In the Italian context, a decisive step forward was taken with the publication of the 2024 Guidelines [76]. This document translates European concerns into practical, field-level instructions for farmers and millers. The project represents a collaborative effort involving FOA (Frantoi Oleari Associati), Unaprol (Consorzio Olivicolo Italiano), and the Universities of Bologna and Udine, promoting a “preventative culture” across the entire production chain. In particular, these guidelines integrate MOH prevention into the HACCP system, identifying the interfaces between machinery and the product as the primary critical control points. Mitigation strategies focus on protecting the olives from exhaust gases and lubricants during harvesting, specifically prohibiting the use of jute bags and mandating documented maintenance of mechanical seals. Within the mill, the directives enforce the exclusive use of food-grade lubricants (NSF-H1) for all moving parts, such as decanter bearings, and the use of hydrocarbon-free detergents, ensuring the entire process is shielded from potential MOH sources.

### 5.2. Effect of Olive Washing

As already highlighted in Section 3.5, olive washing may reduce MOH contamination. To better investigate the effectiveness of washing, Menegoz Ursol et al. [24] analyzed olive samples before and after washing with clean water using a separatory funnel in a controlled laboratory setting. While washing had no appreciable effect on samples with only background MOH levels, it proved highly effective against surface contaminations resulting from harvesting operations, reducing concentrations by 40 to 50%. These laboratory results showed higher and more consistent removal rates compared to those observed under standard mill conditions.

However, it should be noted that these findings are currently based on a limited number of samples (25 olive batches, 14 of which were from the same processing lines) and specific laboratory trials. Therefore, the actual mitigation potential in industrial settings may be lower and more variable, and these results should be considered preliminary. Further large-scale studies are required to validate this effect across different cultivars and diverse contamination scenarios.

Interestingly, while additional water rinses did not improve the removal rate, a subsequent wash with ethanol (which likely removes a portion of the waxy cuticle) proved highly effective, achieving a 95% removal. Although the use of ethanol is impractical for large-scale production due to costs and regulatory constraints. However, these findings suggest that sustainable solutions, such as food-grade surfactants, could be explored to enhance washing efficiency without altering the oil’s composition.

Consistently, the use of dirty recycled water or possible dripping from a pneumatic circuit and lubricated parts during the washing phase can have the opposite effect, introducing new contaminants instead of removing existing ones [56].

### 5.3. The Effect of Refining

Several studies have examined the removal of MOH during oil refining, particularly focusing on deodorization by steam distillation. This process consistently results in a reduction of MOH up to *n*-C_25_–C_30_ [26,60,65,77], with efficiency increasing at higher temperatures [77]. Molecular distillation has also proven effective in controlled environments, achieving up to a 95% reduction of MOH within the *n*-C_25_–C_35_ range [78]. More recently, Gorska et al. [79] demonstrated that both deodorization time and temperature are critical factors for the removal of specific MOAH structures in coconut oil.

In contrast, few studies have specifically addressed the impact of refining on olive oils, often with contrasting results. A study on COPO by Gómez-Coca et al. [25] analyzed seven samples across different refining stages and concluded that the process did not significantly affect the total MOH content. However, since the specific industrial conditions were not reported, it remains unclear whether these results were due to the adoption of particularly mild condition parameters.

Opposing these findings, Menegoz Ursol et al. [24] investigated industrial-scale refining on 6 LOO and 2 COPO samples in two Italian plants. Their results demonstrated a significant effect on the most volatile MOH fraction, in agreement with previous refining studies. MOSH contamination remained fairly constant until the bleaching stage, while deodorization significantly reduced lighter MOH components. Specifically, total MOSH and the *n*-C_10_–C_25_ fraction decreased by approximately 20–30% and 90%, respectively, with MOAH trends mirroring those of MOSH. Samples deodorized under more severe conditions (temperature, duration, and vacuum) showed removal rates consistent with the treatment’s severity. Therefore, while refining has the theoretical potential to act as a mitigation step for shorter-chain hydrocarbons and MOAH, its practical effectiveness in the olive oil industry appears to be highly dependent on specific plant parameters.

Although scarcely investigated, the bleaching step may also contribute to the partial removal of MOAH. Activated carbon, commonly used to remove PAHs originating from the pyrolysis of organic matter, is also capable of partially removing small alkylated PAHs with three or more benzene rings [80]. According to EFSA [2], these compounds represent the most toxic MOAH fraction. Due to their low concentration compared to mono- and di-aromatics, the removal of this highly toxic fraction is generally difficult to detect when analyzing total MOAH. Nevertheless, this effect has been demonstrated in specific OPO samples [71]. Identifying and quantifying this specific fraction remains a priority research area due to its significant toxicological relevance.

**Figure 8:**
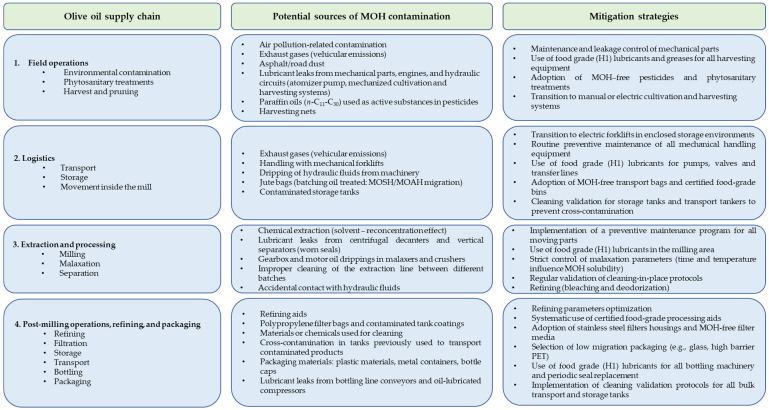
Detailed mapping of the olive oil supply chain, identifying critical entry points for MOH contamination and corresponding mitigation strategies.

Figure 8 illustrates the primary sources of MOH contamination and the corresponding mitigation strategies implemented throughout the olive oil supply chain.

## 6. Conclusions and Perspectives

MOH contamination in olive oils remains a multifaceted challenge that requires a holistic approach across the entire supply chain. While good practices can ensure the virgin olive oils remain free from unacceptable contamination, the OPO sector faces a structural paradox where the extraction process itself amplifies environmental and operational background levels.

Harvesting and mechanical handling are the primary entry points for MOH in virgin oils, largely due to the use of technical-grade lubricants and environmental deposition. In contrast, the high levels in OPO are driven by the exhaustive nature of solvent extraction and the extended storage of pomace.

Mitigation is both possible and effective. The systematic adoption of GAP and GMP, such as switching to NSF H1 food-grade lubricants, has already shown a measurable impact. In this context, a comprehensive review focusing on the role of lubricants in MOH contamination and the latest mitigation strategies is currently in preparation and will be published shortly to provide further guidance.

Furthermore, technical interventions like effective olive washing can provide an additional buffer. Research into food-safe surfactants or optimized washing machinery could offer a sustainable way to clean raw materials before extraction.

The recent EN ISO 20122:2024 standard represents a milestone in ensuring data reliability. However, the persistence of biogenic interferences (e.g., olefins) continues to demand high expertise and advanced cleanup procedures to avoid overestimation.

Looking ahead, the sector must evolve in multiple directions. As toxicological concerns shift toward specific MOAH fractions (≥3R), analytical research must prioritize enrichment techniques to lower LOQs for these genotoxic compounds, moving beyond the simple hump quantification.

A definitive regulatory framework with specific limits for different oil categories is essential. This would provide the industry with clear targets and incentivize the complete phase-out of MOH-based products in favor of synthetic or bio-based alternatives.

To avoid the phasing out of OPO, maximum limits should be reconsidered by accounting for the reconcentration effect. Alternatively, promoting more efficient physical extraction technologies or the sustainable use of olive pomace. This approach would help make the extraction of residual oil unprofitable.

In conclusion, while the olive oil industry has made significant strides in awareness and mitigation, the goal of “MOH-free” production requires continued investment in both analytical precision and preventive technology to maintain the high-quality status of this pillar of the Mediterranean diet.

## Figures and Tables

**Table 1:** MOSH and MOAH content (*n*-C_10_-C_35_ fraction) in EVOOs of different geographical origins sampled in 2018. Values are expressed in mg/kg; LOQ = 1.0 mg/kg (Adapted from [21]).

Origin	N° of Samples	MOSH *n*-C_10_-C_35_ (mg/kg)					MOAH *n*-C_10_-C_35_ (mg/kg)				
		>LOQ (%)	Min	Max	Mean	Median	>LOQ (%)	Min	Max	Mean	Median
Italy	850	100	1.0	64	9.7	7.6	25	1.0	14	2.5	1.9
Greece	107	100	1.7	75	18	14	50	1.1	13	3.9	2.9
Spain	26	100	2.0	58	10	6.1	19	1.5	12	4.9	4.9
UE	152	99	1.0	87	11	7.6	34	1.0	12	3.5	2.3
Non-UE	64	97	1.0	26	4.4	2.5	4.7				

**Table 2:** Summary of total MOSH and MOAH (*n*-C_10_-C_50_) concentrations (mg/kg) reported in literature (2019–2025) for different olive oil categories. Data include sample origin, analytical methods, LOQ, and sample size. Off-line: SFE-GC-FID; Online: LC-GC-FD; MAS: microwave-assisted saponification; Epox: epoxidation; Mdn: median. EVOO: extra virgin olive oil; LOO: *lampante* olive oil; ROO: refined olive oil; OO: olive oil; COPO: crude olive pomace oil; ROPO: refined olive pomace oil; OPO: olive pomace oil.

Olive Oil Sample Type	Origin	Method of Analysis	Sample Preparation	LOQ (mg/kg)	N° of Samples	MOSH *n*-C_10_-C_50_ (mg/kg)				MOAH *n*-C_10_-C_50_ (mg/kg)				Ref.
						Min	Max	Mean	Mdn	Min	Max	Mean	Mdn	
EVOO	-	Off-line	-	1.0	177	2.8	194	13	9.1	1.0	13	2.2	1.5	[21]
EVOO (from mill)	Italy	Online	MAS; epox	0.5	25	0.8	38	8.6	3.7	0.5	12	1.7	0.8	[22]
EVOO (from market)	Italy	Online	MAS; epox	0.5	22	4.8	64	19	14	1.5	6.9	3.4	2.8	[22]
EVOO (from market)	Italy	Online	EN 16995	2.0	9	-	-	-	-	<2.0	2.7	-	-	[23]
LOO	Italy	Online	MAS; epox	0.5	6	5.9	17	11	9.7	1.2	4.9	2.4	1.9	[24]
ROO (deodorized)	Italy	Online	MAS; epox	0.5	6	7.1	28	9.3	9.7	1.1	3.7	2.0	1.6	[24]
ROO	-	Off-line	-	1.0	97	3.7	192	16	9.2	<1.0	25	-	-	[21]
OO (from market)	Italy	Online	MAS; epox	0.5	11	12	88	33	28	1.9	5.2	3.2	2.9	[24]
COPO	Spain	Online	Epox	1.0	10	58	520	216	219	1.8	115	51	57	[25]
ROPO	Spain	Online	Epox	1.0	7	72	547	233	219	6.5	131	59	61	[25]
OPO (from market)	Spain	Online	Epox	1.0	51	33	205	106	94	2.2	55	13	11	[25]
OPO (from market)	Italy	Online	Epox	2.0	12	93	296	170	150	14	67	39	31	[24]

## Data Availability

No new data were created or analyzed in this study. Data sharing is not applicable to this article.
